# Requirement for interleukin-1 to drive brain inflammation reveals tissue-specific mechanisms of innate immunity

**DOI:** 10.1002/eji.201444748

**Published:** 2014-11-24

**Authors:** James A Giles, Andrew D Greenhalgh, Claire L Davies, Adam Denes, Tovah Shaw, Graham Coutts, Nancy J Rothwell, Barry W McColl, Stuart M Allan

**Affiliations:** 1 Faculty of Life Sciences, University of ManchesterManchester, UK; 2 Centre for Research in Neuroscience, Montreal General Hospital, McGill University Health CenterMontreal, Quebec; 3 The Roslin Institute & R(D)SVS, University of EdinburghEaster Bush, Midlothian, UK

**Keywords:** innate immunity, interleukin-1 (IL-1), neuroinflammation, neutrophil

## Abstract

The immune system is implicated in a wide range of disorders affecting the brain and is, therefore, an attractive target for therapy. Interleukin-1 (IL-1) is a potent regulator of the innate immune system important for host defense but is also associated with injury and disease in the brain. Here, we show that IL-1 is a key mediator driving an innate immune response to inflammatory challenge in the mouse brain but is dispensable in extracerebral tissues including the lung and peritoneum. We also demonstrate that IL-1α is an important ligand contributing to the CNS dependence on IL-1 and that IL-1 derived from the CNS compartment (most likely microglia) is the major source driving this effect. These data reveal previously unknown tissue-specific requirements for IL-1 in driving innate immunity and suggest that IL-1-mediated inflammation in the brain could be selectively targeted without compromising systemic innate immune responses that are important for resistance to infection. This property could be exploited to mitigate injury- and disease-associated inflammation in the brain without increasing susceptibility to systemic infection, an important complication in several neurological disorders.

## Introduction

Inflammation is an integral component of the innate immune response providing rapid host defense against infection. Nonmicrobial stimuli (e.g. cell death, hypoxia/ischaemia) can also trigger inflammation, a response that in many conditions is implicated in the exacerbation of tissue injury [1]. Identifying how to contain deleterious effects of inflammation during injury, without compromising host defense to infection, is essential.

A hallmark of an innate immune response is the accumulation of neutrophils in inflamed tissue [2]. All vascularized tissues are able to mount neutrophil-rich innate immune responses if given appropriate stimulation. This includes the brain where we, and others, have shown that dense neutrophil invasion occurs in response to injury or infection [3, 4]. Despite these similarities, it is unclear whether the molecular mechanisms underpinning a conserved innate immune response such as neutrophil recruitment are comparable across tissues.

Interleukin-1 (IL-1)α/β are proinflammatory cytokines considered as key orchestrators of innate immune responses [5]. Production of IL-1 is induced by triggers associated with injury or infection, including microbial ligands and damage-associated molecular patterns [1]. IL-1 induces cytokines, chemokines, growth factors, and vascular adhesion molecules which together co-ordinate neutrophil trafficking and survival [5]. Our previous work has shown the pathologic involvement of IL-1 in neuroinflammatory conditions such as stroke [6], which in part is mediated by neutrophil-driven neurotoxicity [3]. We have also demonstrated the therapeutic potential of anti-IL-1 approaches for treating acute injury to the brain [7–9]; however, a limitation of such an approach is the potential for increasing vulnerability to infection through systemic suppression of innate immunity.

Here we compare the involvement of IL-1 in driving innate immune responses in multiple tissues including the brain. Our results show that the brain is uniquely dependent on IL-1 for mounting an innate immune reaction.

## Results and discussion

### Differential requirements for IL-1 in driving brain versus systemic innate immune responses

**Figure 1:**
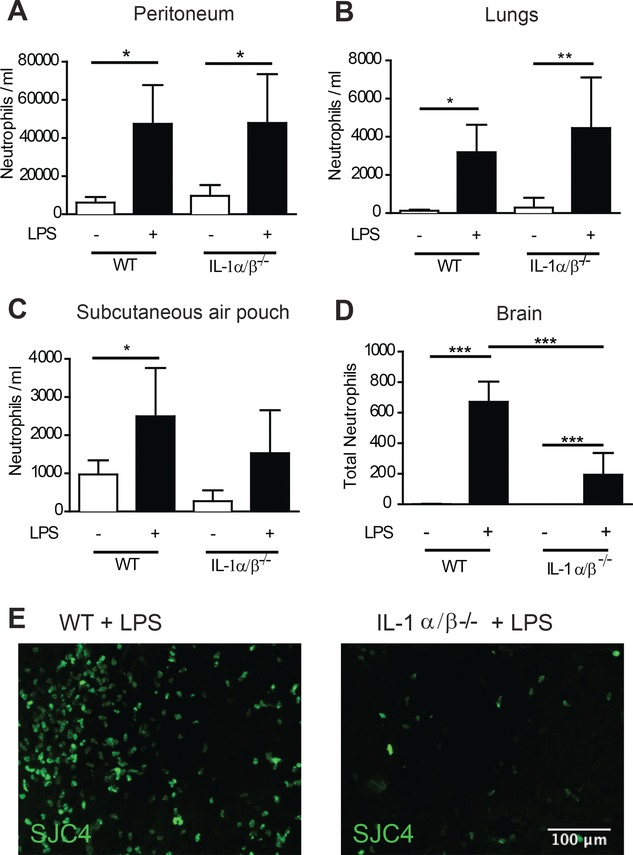
Brain-specific role of IL-1 during the innate immune response (A–E). The innate immune response was triggered by LPS challenge in four different tissues, in WT or IL-1α/β^−/−^ mice and neutrophil accumulation was determined by flow cytometry in (A) the peritoneum, (B) lungs, (C) subcutaneous air pouch or (D) by immunostaining in the brain. (E) Representative (*n* = 5 mice per group) immunofluorescence images of SJC4^+^ neutrophils in brain parenchyma after LPS injection in WT (left panel) and IL-1α/β^−/−^ (right panel) mice, respectively. *p < 0.05, *p* ** < 0.01, *** *p* < 0.0001; two-way ANOVA with Bonferroni correction. Data are presented as mean + SD, *n* = 5 mice per group from a single experiment. Scale bar = 100 μm.

To determine if IL-1 is required to drive innate immune responses in different tissues we stimulated inflammation with the bacterial endotoxin, LPS, in mice deficient in both IL-1 ligands (IL-1α/β^−/−^) or wild-type controls. We used the accumulation of neutrophils as a measure of the intensity of the inflammatory reaction and assessed at timepoints coinciding with peaks of neutrophil influx established previously for the respective tissues. LPS stimulated significant increases in neutrophil accumulation in lavage fluid from the peritoneum, lung, and subcutaneous air-pouch (Fig.1A–C). Similar responses were observed in IL-1α/β^−/−^ mice indicating that IL-1 actions are dispensable for driving cellular recruitment in multiple extracerebral tissues. In contrast, neutrophil recruitment to the brain in response to intrastriatal injection of LPS was significantly attenuated (∼70%) in IL-1α/β^−/−^ mice (Fig.1D, E). These data suggest fundamental differences in the requirement for IL-1 in regulating a key component of innate immune responses in the brain in comparison to extracerebral tissues. To our knowledge, this is the first unequivocal demonstration that IL-1 controls neutrophilic inflammation differently in the brain in comparison to systemic tissues. We showed previously that IL-1 deficiency [10] or administration of IL-1 receptor antagonist (IL-1Ra) [7] reduces neutrophil recruitment to the brain in response to cerebral ischaemia. Alongside the present data, these studies suggest IL-1 is a key driver of innate immune cell recruitment to the brain in response to both microbial ligands and sterile stimuli. Although we cannot be certain the pattern of IL-1 dependence would apply to stimuli other than LPS, our data are consistent with previous studies showing that IL-1 was dispensable for innate immune responses to various microbial-derived triggers in other extracerebral tissues [11–13]. In contrast, IL-1 is a crucial mediator of the response to sterile inflammatory stimuli such as turpentine [14] and necrotic cell preparations [15] outside the brain.

### Tissue-specific activation of IL-1 or compensatory pathways do not underlie brain dependence on IL-1

A potential reason for the brain-specific dependence on IL-1 could be that LPS challenge triggers a different profile of inflammatory mediators in the brain, for example, a response more restricted to activating the IL-1 pathway relative to extracerebral sites. Measurement of a range of cytokines and chemokines showed that LPS induced a similar profile of inflammation across all tissue sites (Supporting Information Fig. 1). LPS induced a significant increase in the concentration of IL-1α (Supporting Information Fig. 1A) and IL-1β (Supporting Information Fig. 1B) in all tissues (with the exception of IL-1α in the peritoneum). These data show that the unique requirement for IL-1 during brain inflammation is not because of tissue differences in the capability to produce IL-1 in response to LPS. The cytokine tumor necrosis factor-α can compensate under inflammatory conditions where there is deficiency of IL-1 [12]. LPS induced a significant increase in TNF-α levels in the brain and in extracerebral tissues (Supporting Information Fig. 1F) suggesting that the reliance on IL-1 in the brain is not because there is a brain-specific inability to initiate alternative/parallel pathways such as via induction of TNF-α. Indeed, in the brain, TNF-α levels in response to LPS were significantly greater in IL-1α/β^−/−^ compared with wild-type mice reinforcing that the brain can activate compensatory pathways to IL-1. Overall, it therefore appears that the molecular profiles of inflammation induced by LPS are similar in the brain and systemic tissues.

### IL-1α mediates the brain-specific requirement for IL-1 in driving innate immunity

**Figure 2:**
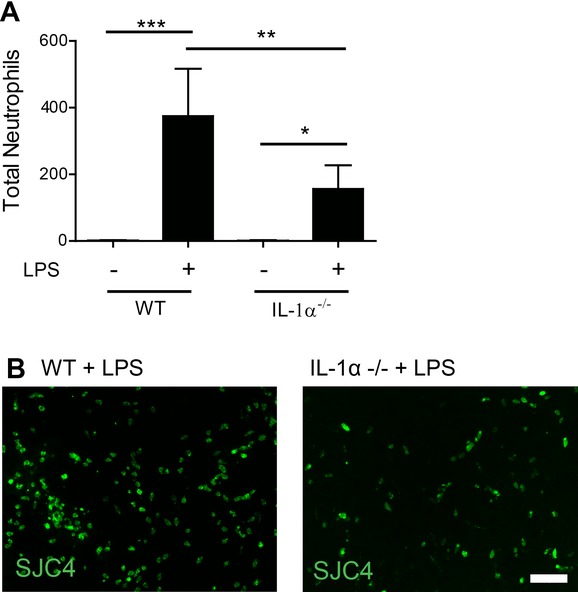
Neutrophil infiltration into brain tissue is mediated by the interluekin-1α ligand. (A) Neutrophil infiltration to brain parenchyma induced by brain injection of LPS in WT and IL-1α^−/−^ mice was determined by quantitative immunstaining. (B) Representative (*n* = 5 mice per group) immunofluorescence images of SJC4^+^ neutrophils in brain parenchyma after LPS injection in WT (left panel) and IL-1α^−/−^ (right panel) mice, respectively. *p < 0.05, p** < 0.01, ***p < 0.0001; two-way ANOVA with Bonferroni correction. Data are presented as mean + SD, *n* = 5 mice per group from a single experiment. Scale bar = 50 μm.

**Figure 3:**
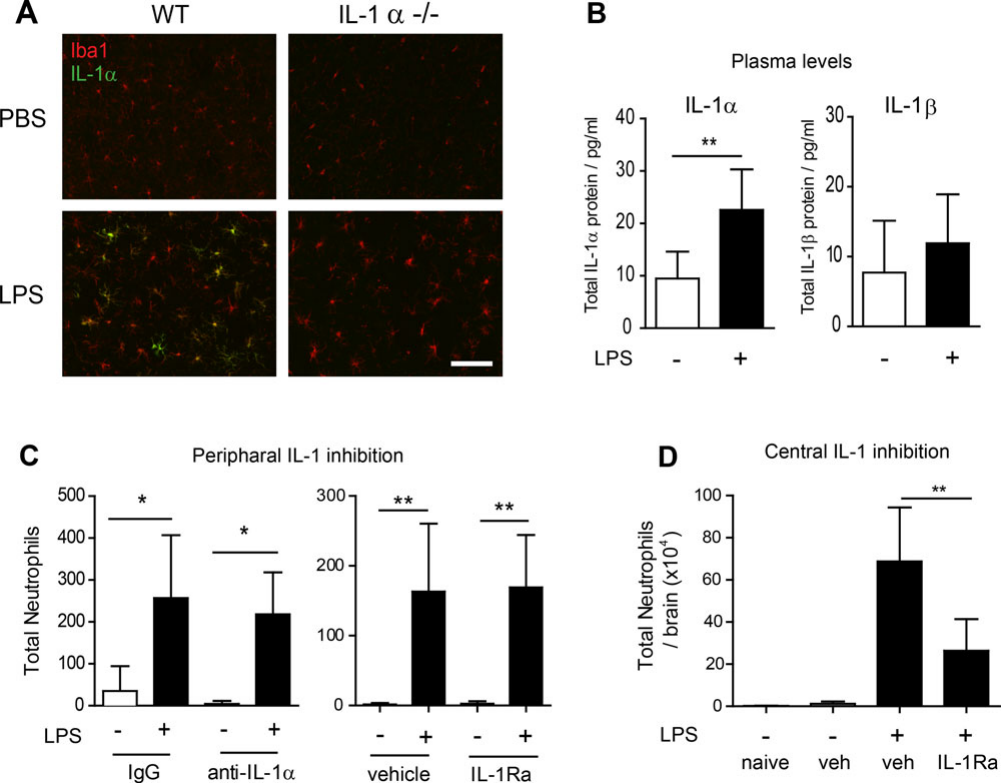
Brain microglia are the likely source of IL-1 required for neutrophil recruitment. (A) Representative images (*n* = 5 mice per group) of IL-1α co-localisation with Iba1-positive microglia in the brain 24 h after LPS injection in WT (left panels) and IL-1α^−/−^ (right panels) mice was determined by immunostaining. (B) IL-1α and IL-1β concentrations in the blood plasma of mice 6 h after brain LPS injection were determined by cytometric bead array. (C) Neutrophil infiltration into the brain following treatment with specific IL-1a neutralizing antibody (left panel), or the interleukin-1 receptor antagonist (IL-1Ra) (right panel) was determined by quantitative immunostaining. (D) Neutrophil accumulation in the brain following simultaneous intracerebral injection of LPS with IL-1Ra or vehicle was determined by flow cytometry. *p < 0.05, p** <0 .01, two-way ANOVA with Bonferroni correction. Data are presented as mean + SD, *n* = 5 mice per group from a single experiment. Scale bar = 50 μm

Our recent data implicated IL-1α as the key IL-1 agonist driving cerebrovascular inflammation [16]. We next determined the functional contribution of IL-1α to the IL-1-dependent innate immune reaction induced by intracerebral LPS challenge. Neutrophil recruitment to the brain was significantly reduced in IL-1α^−/−^ mice (Fig.2). The magnitude of the effect was similar to that observed in IL-1α/β^−/−^ mice suggesting that IL-1α could be the dominant ligand responsible for the dependence on IL-1. However we cannot entirely exclude a role for IL-1β, particularly given that intracerebral IL-1β administration or overexpression is capable of inducing neutrophil infiltration to the brain and associated chemokines [17, 18]. Intracerebral LPS caused changes in microglial morphology and Iba1 immunostaining, consistent with microglial activation e.g. hypertrophy of cell soma, retraction of processes (Fig.3A). IL-1α expression was markedly induced on activated microglia in the hemisphere ipsilateral to injection in wild-type mice but was completely absent in IL-1α^−/−^ mice (although microglia retained an activated morphology) (Fig.3A). Although IL-1α and IL-1β have overlapping roles under many inflammatory conditions, recent evidence suggests that IL-1α is the predominant ligand mediating inflammation early after inflammatory insults to the brain, such as in response to cerebral ischaemia [19, 20]. Outside the brain, sterile inflammatory stimuli such as necrotic cell preparations that contain damage-associated molecular patterns trigger robust inflammatory responses including neutrophil recruitment that are dependent on IL-1α [15]. Thus, unlike the brain, where the present and recent data point to a universal dependence on IL-1, in extracerebral tissues there appears to be a context-dependent requirement for IL-1 depending on whether the stimulus is microbial or sterile.

### Intracerebral sources of IL-1 are responsible for the brain-specific requirement for IL-1

Induction of IL-1α was not limited to the brain after intracerebral LPS as we observed a significant increase in IL-1α (but not IL-1β) concentration in blood plasma 6 h after intracerebral LPS injection (Fig.3B). This induction was absent in platelet-depleted mice indicating platelets as a key systemic source of IL-1α in this model (Supporting Information Fig. 2) and responsive to intracerebral LPS indirectly (e.g. as part of systemic acute phase response) or directly via platelet expression of TLR4 [21]. We sought to determine the relative importance of intra- and extracerebral sources of IL-1 in driving neutrophil recruitment to the brain in response to intracerebral LPS challenge. In this model of brain inflammation there is negligible disruption to the blood–brain barrier as shown by minimal leakage of endogenous circulating IgG in the brain that is comparable in veh- and LPS-injected mice. This restricts agents administered systemically from entering the brain parenchyma and provides a capability to selectively target IL-1 produced outside the brain. We first administered an anti-IL-1α neutralizing antibody systemically to mice prior to LPS challenge. Lack of penetration of the BBB was confirmed by the absence of immunostaining for rat IgG in anti- IL-1α IgG- or control IgG-injected mice (Supporting Information Fig. 3A–B). Neutrophil accumulation in the brain in response to LPS challenge was similar in both anti- IL-1α and control IgG-treated mice (Fig.3C). Given that the distribution of the antibodies was contained systemically these data implicate that IL-1α derived from cells in the brain is the key source driving neutrophil recruitment. We substantiated this finding using an alternative approach to selectively target systemic IL-1 via intraperitoneal administration of IL-1Ra. As above, we confirmed that IL-Ra did not reach the brain parenchyma (Supporting Information Fig. 3C) Accumulation of neutrophils in the brain in response to intracerebral LPS challenge was not altered in mice administered IL-1Ra systemically (Fig.3C) which further supports that the brain compartment is the critical source of IL-1. In contrast, IL-1Ra administered directly into the brain significantly attenuated neutrophil accumulation in response to intracerebral LPS (Fig.3D). As described above intracerebral LPS challenge induced expression of IL-1α which was exclusively localized to microglia. Thus, microglial-derived IL-1 appears to be the key source responsible for the unique dependence on IL-1 in this model.

### Concluding remarks

Immunosuppression and increased susceptibility to infection are potentially unwanted adverse effects of immunomodulatory treatments targeting injury or disease-associated inflammation. This is a particular issue for brain disorders, most notably acute injuries such as stroke where systemic infection is a common complication and leading cause of mortality [22]. The ability to selectively modify pathologic inflammation in the injured brain without overly suppressing systemic immunity would be advantageous. Our present data highlight that IL-1 is a target that could fulfil these criteria. Neutrophil recruitment is a hallmark of innate immune responses and essential for effective defense against bacterial infection [2]. We and others have shown previously that neutrophils contribute to exacerbation of injury in the brain [3, 4]. Our present data reveal that blocking IL-1α activity can markedly reduce neutrophil recruitment to the inflamed brain but, in contrast, IL-1 is completely dispensable for neutrophil recruitment to multiple extracerebral tissues. This suggests that anti-IL-1 interventions, at least given acutely, may have the capacity to limit injury-amplifying inflammation in the brain without markedly weakening systemic innate immunity needed for host defense. In support, recent clinical trials testing the safety of recombinant human IL-1Ra after stroke [9] or subarachnoid hemorrhage [23] reported a lower than average incidence of infection. Moreover, there is some indication that IL-1Ra may actually protect against systemic immunosuppressive responses to stroke [24].

In conclusion, we have demonstrated dependence on IL-1 for driving an innate immune response to inflammatory challenge in the brain that contrasts with IL-1-independence in extracerebral tissues. We believe these properties could be exploited therapeutically to minimize systemic immunosuppressive complications of anti-inflammatory therapy in neurological disorders.

## Materials and methods

### Animals

Experiments were performed on male 8–10 week-old C57Bl/6J, IL-1αβ^−/−^ and IL-1α^−/−^ mice and adhered to the UK Animals (Scientific Procedures) Act 1986.

### Inflammatory challenge

#### Peritoneal inflammation model

Mice were injected intraperitoneally with 1 mg/kg LPS from *Escherichia coli* O127:B8 (Sigma-Aldrich, Dorset, UK) or vehicle (PBS) in a volume of 8 mL/kg. Peritoneal lavage was performed at 6 h using 5 mL PBS containing 0.1% BSA and 1 mM EDTA.

#### Broncho-alveolar inflammation model

Mice were exposed to aerosolized LPS (2 mg/mL) or vehicle (saline) for 20 min and broncho-alveolar lavage performed after 6 h with 1 mL of PBS containing 0.1% BSA and 1 mM EDTA.

#### Air pouch inflammation model

Dorsal air pouches were created in conscious mice as described previously [25]. At day 7, 1 mL of LPS (1 mg/mL) or vehicle (PBS) was injected into the air pouch. After 6 h air pouch lavage was performed using 4 mL PBS, with 0.1% BSA and 1 mM EDTA.

#### Cerebral inflammation model

Animals were anesthetized with isoflurane (3%) in O_2_ (200 mL/min) and N_2_O (400 mL/min) and craniectomy performed. Mice were injected intracerebrally with 1 μL LPS (4 mg/mL), via a glass microneedle (co-ordinates from bregma: anterior–posterior −0.0 mm, lateral −2.0 mm, ventral −2.5 mm. Rate = 0.5 μL/min). Recombinant human IL-1Ra (10 μg) or placebo were coinjected with LPS. Mice were transcardially perfused with saline at 6 h and brain tissue collected for CBA analysis or perfuse-fixed (saline followed by paraformaldehyde 4%) at 24 h for tissue sectioning.

### Flow cytometry

Lavage fluid cell suspensions (200 μL) were incubated for 20 min with rat anti-mouse CD16/CD32 to block nonspecific Fc binding then stained with anti-mouse Ly-6G (1A8) for 30 min. Brain cell suspensions were prepared by enzymatic digestion and density gradient centrifugation and cells incubated with anti-CD16/CD32 before staining with anti-mouse Ly6G-Pacific Blue. Absolute cell counts were determined by the addition of 50 μL fluorescent counting beads (Invitrogen, Paisley, UK). Flow cytometry was performed on a CyAn™ ADP Flow Cytometer (Dako UK Ltd, Ely, UK) or BD Fortessa (BD Biosciences USA) and data was analyzed using Summit 4.0 software.

### Immunostaining

Immunohistochemistry was performed on coronal brain sections using standard protocols. Anti-neutrophil (SJC4, rabbit anti-mouse) primary antibody (1:50 000; kindly provided by Drs. D Anthony and S Campbell, University of Oxford, UK) was used to stain for neutrophils and anti-Iba1 for microglia. Neutrophil numbers were quantified in three regions of interest (cerebral cortex, injection site, and ventral striatum) using a 10 × 10 mm graticule at 20× magnification and expressed as the sum of the counts in each region of interest.

### Cytometric bead array

Cytokine concentrations in plasma and lavage samples were determined using mouse-specific CBA flex sets (BD Pharmingen, Oxford, UK) following manufacturer's guidelines with an optimized 1:5 dilution of concentrations. Acquisition was undertaken using a BD FACSArray™ Bioanalyzer System (BD Biosciences, Oxford, UK), and results determined using FCAP Array™ software (Soft Flow, Burnsville, Minnesota, USA).

### Systemic IL-1 interventions

Mice were injected i.p. with anti-IL-1α or IgG isotype control (4 mg/kg), 24 h before intracerebral injection of LPS. IL-1Ra (10 mg/kg) or saline was administered i.p. 1 h before and 2 h after LPS injection. Doses were chosen based on ND50 and our previous data on cytokine responses to LPS.

### Statistics

Data are expressed as mean (± SD). Differences between groups were analyzed using two-way ANOVA with post-hoc Bonferroni correction. Differences were considered statistically significant at *p* < 0.05. Sample sizes were estimated using power analysis on data from pilot experiments. Mice were randomized to treatment groups.

## Abbreviations

IL-1Ra: IL-1 receptor antagonist
