# Supplementary material for: Requirement for interleukin-1 to drive brain inflammation reveals tissue-specific mechanisms of innate immunity
Source: Eur J Immunol. 2014 Nov 24;45(2):525–30. doi: 10.1002/eji.201444748 (PMC4357393; doi:10.1002/eji.201444748)
Supplement: Supplementary file 1 [file eji0045-0525-sd1.pdf]

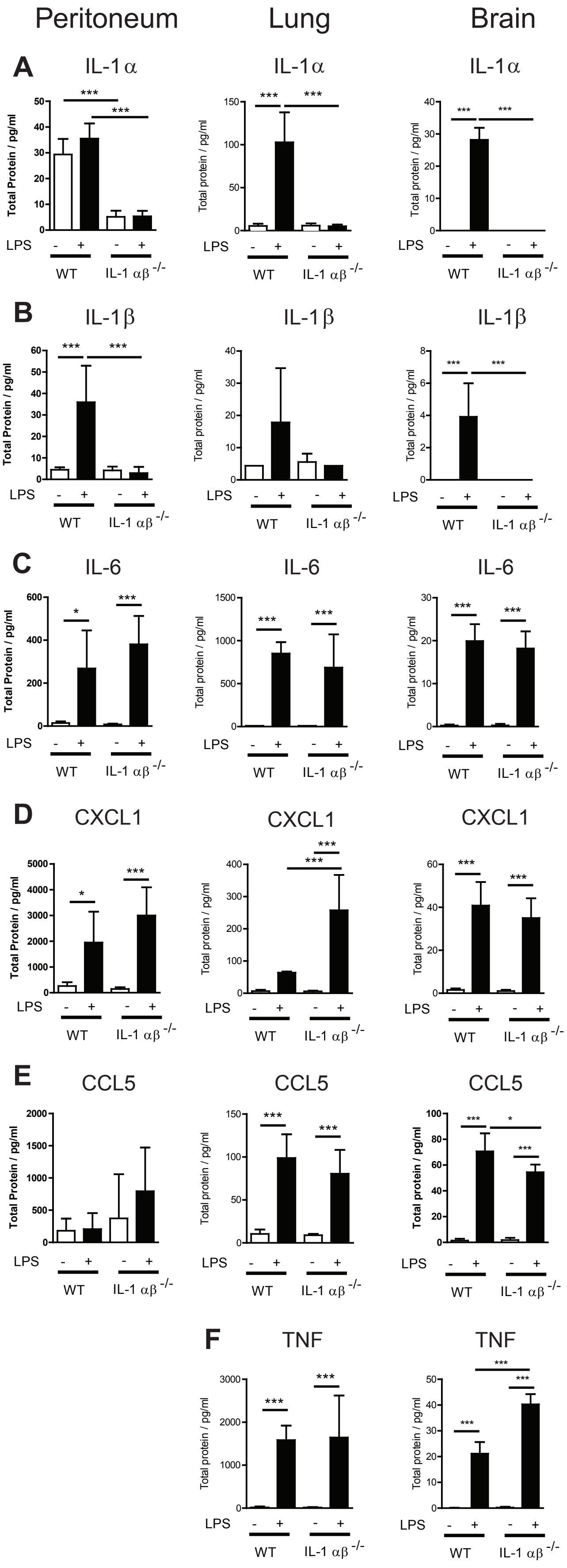

Peritoneum
Lung
Brain
A
IL-1α
B
IL-1β
C
IL-6
D
CXCL1
E
CCL5
F
TNF
Total Protein / pg/ml
Total protein / pg/ml
LPS
-
+
WT
IL-1 αβ -/-
***
*

Supplemental figure 1

Brain dependence on IL-1 is not related to tissue-specific differences in activating IL-1 or established compensatory pathways. A panel of inflammatory cytokines; IL-1α, IL-1β, IL-6 and TNF and chemokines; CXCL1 and CCL5 were assessed in peritoneum (left column), lung (middle column) and brain (right panel) after injection of LPS. LPS induced a significant increase in the concentration of IL-1α (**A)** and IL-1β (**B)** in all tissues (with the exception of IL-1α in the peritoneum). LPS also induced an increase in the majority of mediators across all tissue beds, which was largely unaffected by the absence of IL-1 in IL-1α/β $^{-/-}$ mice. * $p<0.05$, p**<0.01, *** $p<0.0001$; two-way ANOVA with Bonferroni correction, n=5. Data are presented as mean ± SD.

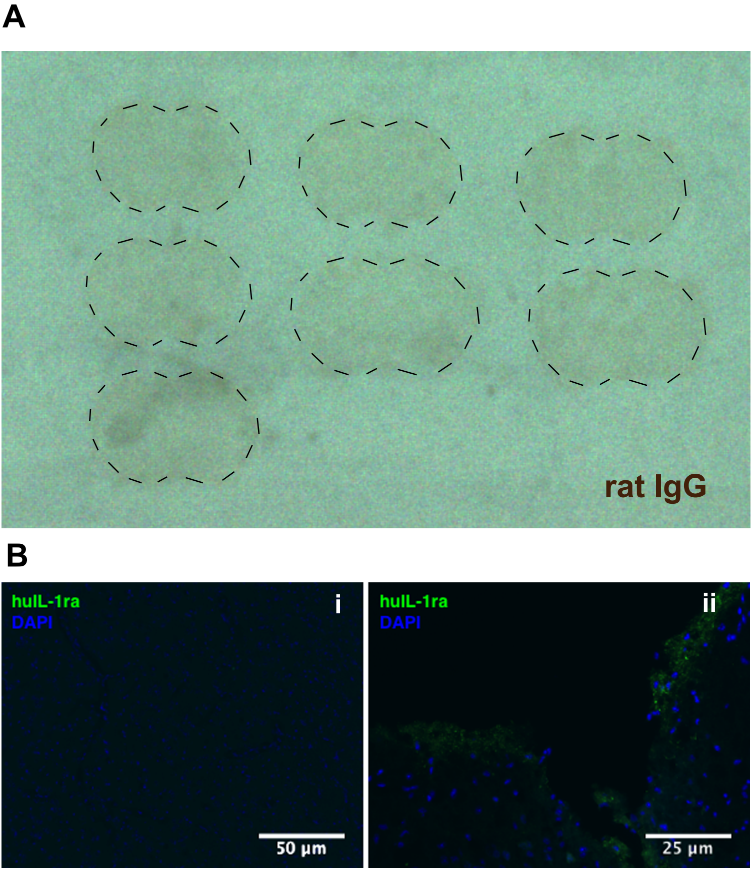
A
rat IgG
B
huIL-1ra
DAPI
i
50 µm
huIL-1ra
DAPI
ii
25 µm

Supplemental figure 2

Systemically administered IL-1 neutralising antibodies/antagonists do not enter brain parenchyma and provide the capability to selectively target IL-1 produced outside the brain. Representative images confirm lack of penetration of the blood-brain barrier (BBB) by the absence of immunostaining for rat IgG in anti- IL-1α IgG (i.p) injected mice **(A)**; and the absence of immunostaining for IL-1Ra in the parenchyma of recombinant human-IL-1Ra (huIL-1Ra) (i.p) injected mice **(Bi).** Right panel **(Bii)** shows huIL-1Ra staining in areas of BBB breakdown at the site of craniectomy.
