# Supplementary material for: Requirement for interleukin-1 to drive brain inflammation reveals tissue-specific mechanisms of innate immunity
Source: Eur J Immunol. 2014 Nov 24;45(2):525–30. doi: 10.1002/eji.201444748 (PMC4357393; doi:10.1002/eji.201444748)
Supplement: Supplementary file 2 [file eji0045-0525-sd2.pdf]

European Journal of Immunology

Peer review correspondence

Requirement for interleukin-1 to drive brain inflammation reveals tissue-specific mechanisms of innate immunity

James A. Giles, Andrew D. Greenhalgh, Claire L. Davies, Adam Denes, Tovah Shaw, Graham Coutts, Nancy J. Rothwell, Barry W. McColl and Stuart M. Allan

Corresponding author: Barry W. McColl, The Roslin Institute & R(D)SVS, University of Edinburgh, Easter Bush, Midlothian, United Kingdom

---

Review Timeline:	Submission date:	15 April 2014
	First editorial decision:	2 June 2014
	First revision received:	29 August 2014
	Second editorial decision:	19 September 2014
	Second revision received:	30 September 2014
	Accepted:	22 October 2014

Handling Executive Committee member: Prof. Britta Engelhardt

Please note that the correspondence below does not include the standard editorial instructions regarding preparation and submission of revised manuscripts, only the scientific revisions requested and addressed.

First Editorial Decision – 2 June 2014

Dear Dr. McColl,

Please accept my apologies for the delay in processing the review of your Manuscript ID eji.201444748 entitled "Exclusive requirement for interleukin-1 in driving brain inflammation reveals tissue-specific mechanisms of innate immunity" which you submitted to the European Journal of Immunology. We were awaiting two delayed reports; indeed one report was never submitted. However, we feel the comments of the two referees are sufficient for a decision; the comments of the referees are included at the bottom of this letter. You should know that this is not the usual peer review turnaround time for the Journal.

A revised version of your manuscript that takes into account the comments of the referees will be reconsidered for publication.

You should also pay close attention to the editorial comments included below. *In particular, please edit your figure legends to follow Journal standards as outlined in the editorial comments. Failure to do this will result in delays in the re-review process.*

Please note that submitting a revision of your manuscript does not guarantee eventual acceptance, and that your revision will be re-reviewed by the referees before a decision is rendered.

If the revision of the paper is expected to take more than three months, please inform the editorial office. Revisions taking longer than six months may be assessed by new referees to ensure the relevance and timeliness of the data.

Once again, thank you for submitting your manuscript to European Journal of Immunology and we look forward to receiving your revision.

Yours sincerely,
Karen Chu

On behalf of Prof. Britta Engelhardt

Dr. Karen Chu
Editorial Office
European Journal of Immunology
e-mail: ejied@wiley.com
www.eji-journal.eu

****

Reviewer: 1
Comments to the Author
This study by Giles et al. shows that IL-1 is a critical mediator driving neutrophil infiltration in the mouse brain after challenge with the bacterial endotoxin LPS, but not in peripheral tissues such as the lung and peritoneum. In particular, the authors found that microglia-derived IL-1alpha is responsible for neutrophil recruitment following intracerebral LPS injection. From these observations, the authors conclude that IL-1-mediated inflammation in the CNS could be selectively targeted without compromising systemic innate immunity against bacterial infection. The study represents a significant advance. However, a number of issues must first be addressed.

Major Points:

1) The title could be more informative of the paper content.

2) It would be helpful if the authors could clarify whether neutrophils infiltrate the CNS following intraperitoneal injection of LPS. According to the results showing that microglia are the main source of IL-1alpha in the inflamed brain, i.p. LPS injection should not lead to neutrophil recruitment into the CNS unless it penetrates the BBB.

3) In order to convincingly conclude that IL-1 is dispensable for innate immunity to microbial-derived triggers in peripheral tissues, I would strongly recommend to test at least one other PAMP. Otherwise, please temper the conclusions of the paper.

4) Please show that neutrophil count is normal under basal conditions in the bone marrow and blood of IL-1a/b-/- and IL-1a-/- mice.

5) Do IL-1a-/- mice express normal levels of IL-1b in normal conditions and after intracerebral LPS injection?

6) The observation that IL-1a levels are increased in the blood plasma at 6 h after intracerebral LPS injection, and that this induction depends on the presence of platelets, is important and intriguing. I would recommend to include these data in the supplementary information section, and briefly comment on why platelets would be responsive to LPS or microglia-derived IL-1a. In other words, please cite the work reporting expression of CD14, TLR4 and IL-1R1 in platelets.

7) Although I understand that this is a short communication and that this is perhaps a lot to ask but it would have been a plus to show that neutrophil infiltration in blocked upon intracerebroventricular delivery of anti-IL-1a antibody or IL-1Ra.

8) Which molecule does the anti-SJC4 antibody recognize exactly? How come the anti-Ly-6G antibody was not used instead?

Typo: IL-1Ra (page 8, 2nd paragraph)

Reviewer: 2
Comments to the Author
The main contention of this paper is that IL-1 is an indispensable driver of neutrophil infiltration in response to LPS in the brain but not in peripheral organs. While it is well established that IL-1 is sufficient for neutrophil infiltration in the brain studies directly comparing the role of IL-1 in LPS-induced neutrophil

European Journal of Immunology

infiltration in different tissues has not, to my knowledge, been performed. The results are relatively clear:, equivalent neutrophil infiltration was observed in wild-type and IL-1alpha/beta knockout in peritonitis, lung and air pouch models of LPS-induced inflammation. However after intracerebral LPS challenge neutrophil infiltration was significantly less in IL-1 alpha/beta knockout mice. Nonetheless, there remains some residual neutrophil infiltration in IL-1 alpha/beta knockout mice and the title suggests that IL-1 is indispensable for the PMN influx, so this is not quite right and should be dealt with both in the phrasing of the title, in the discussion and also in the results sections as I detail below.

Figure 1 – no issues

The authors further argue that the major driver of CNS innate immunity is IL-1 alpha and that microglia are the major provider.

Figure 2 – IL-1alpha-/- mice show less neutrophil infiltration after LPS i.c.. However this decrease is of the order of 50% which doesn't rule out a significant role of IL-1b and since we know from the studies of Anthony/Perry and others that IL-1b is sufficient to induce neutrophil infiltration into the brain, it remains entirely possible that IL-1 has a significant role.

In Figure 3 the authors go on to ask whether the role of IL-1 alpha is central or peripheral, which is valid, but it is strange that they do not investigate whether IL-1b has any role in the observed neutrophil infiltration.

Figure 3 –

A) The authors also show that the LPS-induced hypertrophy still occurs in the IL-1 alpha animals: Do these occur in the alpha/beta knockout animals ? Does IL-1b drive the hypertrophy changes?

B) The fact that central LPS drives a mild peripheral IL-1a response appears to be the justification for assessing the role of systemically applied anti-IL-1 IgG and IL-1ra to assess the role of circulating IL-1a in the LPS induced PMN recruitment in Figure 3C. This seems valid to test, but this seemed unlikely and was perhaps less pressing than testing whether centrally applied IL-1ra/anti-IL-1 IgG block the PMN recruitment and whether blocking IL-1b would do likewise. The authors also indicate that the IL-1a response is absent in platelet depleted animals and this data might be shown?

Given that it is one of the conclusions of the abstract that IL-1 alpha is the dominant IL-1 form in brain innate immunity and this is implied in 2 of the 3 figures in this short communication, I would suggest that the authors should probably demonstrate/refute the contribution of IL-1b to the LPS-induced neutrophil infiltration to the brain.

Supplementary figure 2: Given that the authors take the trouble to investigate whether IgG and IL-1ra do not enter the brain parenchyma, they should show these data at sufficient resolution to examine whether there really is no IgG in the brain; supplementary figure 2A is at very low power, is insufficiently labelled

and (if these pictures are intended to demonstrate lack of IgG labelling), they should demonstrate a positive control, in which they predict blood brain barrier breakdown and in which IgG labelling is successfully demonstrated. The addition of IL-1ra labelling at the site of craniotomy (C) is useful makes the lack of labelling in B credible and the same approach might be used for IgG.

Minor points:

Reference 13 is incomplete (no year)

First revision – authors' response – 29 August 2014

Reviewer: 1

Comments to the Author
This study by Giles et al. shows that IL-1 is a critical mediator driving neutrophil infiltration in the mouse brain after challenge with the bacterial endotoxin LPS, but not in peripheral tissues such as the lung and peritoneum. In particular, the authors found that microglia-derived IL-1alpha is responsible for neutrophil recruitment following intracerebral LPS injection. From these observations, the authors conclude that IL-1-mediated inflammation in the CNS could be selectively targeted without compromising systemic innate immunity against bacterial infection. The study represents a significant advance. However, a number of issues must first be addressed.
Response: We thank the reviewer for their positive comments and provide a point-by-point response below. We draw attention to the inclusion of new data, both in this response (point 4 and 5), and in the manuscript (point 6 and 7).

Major Points:

1) The title could be more informative of the paper content.
Response: We have amended the tile to read "Requirement for interleukin-1 in driving brain inflammation reveals tissue-specific mechanisms of innate immunity" which we believe reflects the key outcomes from the study

2) It would be helpful if the authors could clarify whether neutrophils infiltrate the CNS following intraperitoneal injection of LPS. According to the results showing that microglia are the main source of IL-1alpha in the inflamed brain, i.p. LPS injection should not lead to neutrophil recruitment into the CNS unless it penetrates the BBB.
Response: This is an interesting question however one that we believe is outside the scope of the present study as trafficking of immune cells to sites distant from the inciting stimulus is a markedly different question. LPS given ip is well known to induce inflammatory mediators in the brain and there are some data suggesting neutrophils can be recruited (although their distribution is largely perivascular[1]). We suspect this is because the profile of inflammatory mediators induced in the brain is different when the stimulus is local (intracerebral) or distant (intraperitoneal). The key question in our study was if trafficking of neutrophils to the site local to the inciting stimulus was similar across different tissues.

3) In order to convincingly conclude that IL-1 is dispensable for innate immunity to microbial-derived triggers in peripheral tissues, I would strongly recommend to test at least one other PAMP. Otherwise, please temper the conclusions of the paper.
Response: We agree with the reviewer's comment and have amended the text on p4 to read "Although we cannot be certain the pattern of IL-1 dependence would apply to stimuli other than LPS, our data are

European Journal of Immunology

consistent with previous studies showing that IL-1 was dispensable for innate immune responses to various microbial-derived triggers in other extra-cerebral tissues [11] [12, 13]"

4) Please show that neutrophil count is normal under basal conditions in the bone marrow and blood of IL-1a/b-/- and IL-1a-/- mice.

Response: we have shown previously that steady-state blood neutrophil numbers are unaffected in these mice[2], which we have replicated for the IL-1α/β-/- mice in this study and included here (see figure below)Furthermore, our data show that basal neutrophil numbers in the peritoneum and lung (which harbour these cells normally) are unaffected (Fig 1A,B). This is consistent with the principal role of IL-1 under inflammatory conditions and thus we are confident that effects of IL-1 deficiency in the intracerebral model are not due to underlying differences in systemic neutrophil numbers.

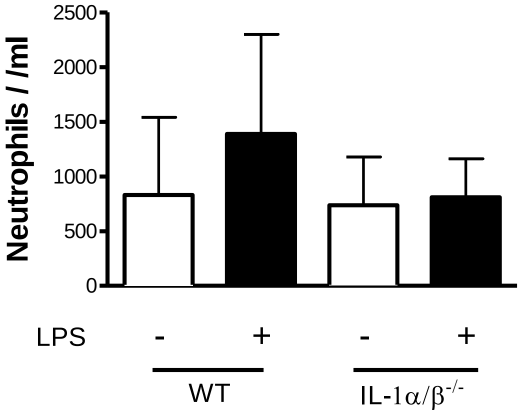

Blood neutrophil numbers were unaffected by genotype in this study, as shown previously[2]

5) Do IL-1a-/- mice express normal levels of IL-1b in normal conditions and after intracerebral LPS injection?

Response: IL-1beta levels are usually undetectable in the naïve brain (e.g. see supplementary figure 1). We have published previously that IL-1beta mRNA levels are not different in the brains of IL-1alpha KO mice[3]. Our unpublished data also show that in response to intracerebral LPS challenge, IL-1beta levels in blood are similar in IL-1alpha KO and WT mice (see figure below). We would not completely rule out that there could be some compensation by IL-1beta in IL-1alpha KO mice however this would mean we actually underestimate the contribution of IL-1alpha in the present study.

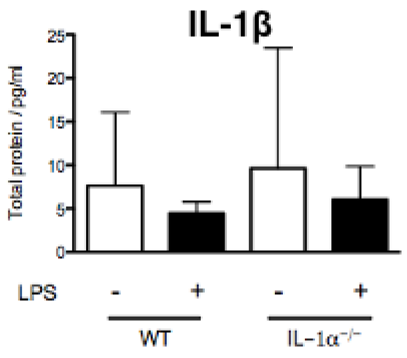

Concentration of IL-1beta in plasma of WT and IL-1alpha KO mice in response to intracerebral LPS challenge.

6) The observation that IL-1a levels are increased in the blood plasma at 6 h after intracerebral LPS injection, and that this induction depends on the presence of platelets, is important and intriguing. I would recommend to include these data in the supplementary information section, and briefly comment on why

platelets would be responsive to LPS or microglia-derived IL-1a. In other words, please cite the work reporting expression of CD14, TLR4 and IL-1R1 in platelets.
Response: We have now included these data in supplemental figure 2. Although it is possible that platelets may respond directly to ic administered LPS we think it is more likely that the inflammation induced in the brain triggers a systemic acute phase response and the induction of platelet IL-1alpha is an indirect mechanism. Given the largely autocrine/paracrine actions of IL-1 ligands we would also not expect that IL-1alpha derived from microglia is directly stimulating platelets in the circulation. We have amended the text of p7 of the manuscript including relevant citation: ”This induction was absent in platelet-depleted mice indicating platelets as a key systemic source of IL-1□ in this model and responsive to intracerebral LPS indirectly (e.g. as part of systemic acute phase response) or directly via platelet expression of TLR4[19]”

7) Although I understand that this is a short communication and that this is perhaps a lot to ask but it would have been a plus to show that neutrophil infiltration in blocked upon intracerebroventricular delivery of anti-IL-1a antibody or IL-1Ra.
Response: We thank the reviewer for this suggestion and include new data in Figure 3D showing that IL-1Ra administered ic significantly reduces neutrophil infiltration to the brain, confirming brain derived IL-1 is responsible for neutrophil infiltration. These data were collected using flow cytometry. We have also added the following text to p8 of the manuscript: “In contrast, IL-1Ra administered directly into the brain significantly attenuated neutrophil accumulation in response to intracerebral LPS (Fig 3). Claire Davies, who performed this experiment, has been added to the list of authors.

8) Which molecule does the anti-SJC4 antibody recognize exactly? How come the anti-Ly-6G antibody was not used instead?
Response: The anti-SJC4 antibody is a polyclonal antiserum raised in rabbits against rat neutrophils. We have extensive experience using this antibody in mice and rats to specifically detect neutrophils by immunohistochemistry and find staining quality is far superior to Ly6G. However, we routinely use Ly6G (1A8 clone) for detecting neutrophils by flow cytometry.

Typo: IL-1Ra (page 8, 2nd paragraph)
Response: We have corrected the typographical error.

Reviewer: 2

Comments to the Author
The main contention of this paper is that IL-1 is an indispensable driver of neutrophil infiltration in response to LPS in the brain but not in peripheral organs. While it is well established that IL-1 is sufficient for neutrophil infiltration in the brain studies directly comparing the role of IL-1 in LPS-induced neutrophil infiltration in different tissues has not, to my knowledge, been performed. The results are relatively clear:, equivalent neutrophil infiltration was observed in wild-type and IL-1alpha/beta knockout in peritonitis, lung and air pouch models of LPS-induced inflammation. However after intracerebral LPS challenge neutrophil infiltration was significantly less in IL-1 alpha/beta knockout mice. Nonetheless, there remains some residual neutrophil infiltration in IL-1 alpha/beta knockout mice and the title suggests that IL-1 is indispensable for the PMN influx, so this is not quite right and should be dealt with both in the phrasing of the title, in the discussion and also in the results sections as I detail below.
Response: We appreciate the reviewer’s comments and have rephrased the manuscript in several places, including the title (see response to reviewer 1), to reflect that IL-1 is a key mediator in brain versus systemic tissues but acknowledging that there is some residual neutrophil infiltration in IL-1 deficient mice.
We would like to draw attention to the inclusion of new data, both in this response and in the manuscript (point 3).

Figure 1 – no issues

Peer review correspondence

European Journal of Immunology

The authors further argue that the major driver of CNS innate immunity is IL-1 alpha and that microglia are the major provider.
Figure 2 – IL-1alpha-/- mice show less neutrophil infiltration after LPS i.c.. However this decrease is of the order of 50% which doesn't rule out a significant role of IL-1b and since we know from the studies of Anthony/Perry and others that IL-1b is sufficient to induce neutrophil infiltration into the brain, it remains entirely possible that IL-1 has a significant role.
Response: We agree with the reviewer that a role for IL-1beta cannot be completely excluded as we state on p6 of the manuscript: "The magnitude of the effect was similar to that observed in IL-1α/β-/- mice suggesting that IL-1α is the predominant ligand responsible for the dependence on IL-1, although we cannot entirely exclude a role for IL-1β".

In Figure 3 the authors go on to ask whether the role of IL-1 alpha is central or peripheral, which is valid, but it is strange that they do not investigate whether IL-1b has any role in the observed neutrophil infiltration.
Figure 3 –
A) The authors also show that the LPS-induced hypertrophy still occurs in the IL-1 alpha animals: Do these occur in the alpha/beta knockout animals ? Does IL-1b drive the hypertrophy changes?
Response: Our unpublished data (see figure below) show that the morphological signs of microglial activation including hypertrophy still occur in IL-1alpha/beta KO. Our unpublished flow cytometry data also show that the increase in CD45 (gross marker of activation) intensity on microglia after ic LPS challenge is not affected by co-administration of IL-1Ra directly into the brain (see figure below). These data suggest that IL-1 in general is dispensable for general signs of microglial activation, which we speculate may be driven by signals upstream of IL-1 induction.

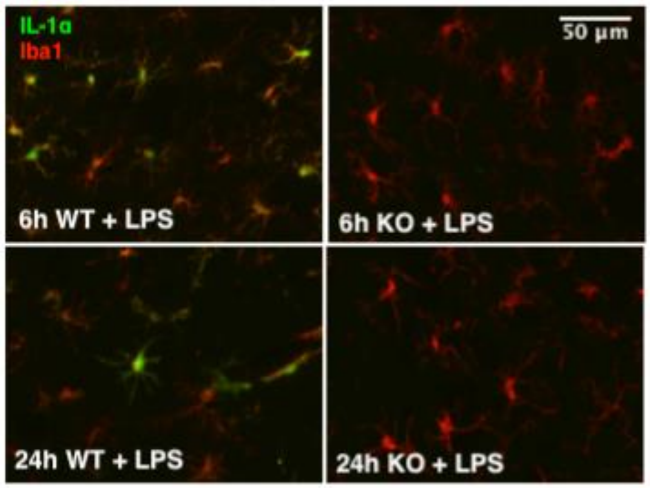

Morphological signs of microglial activation (e.g. hypertrophy) were similar in IL-1alpha/beta KO and WT mice after ic LPS challenge

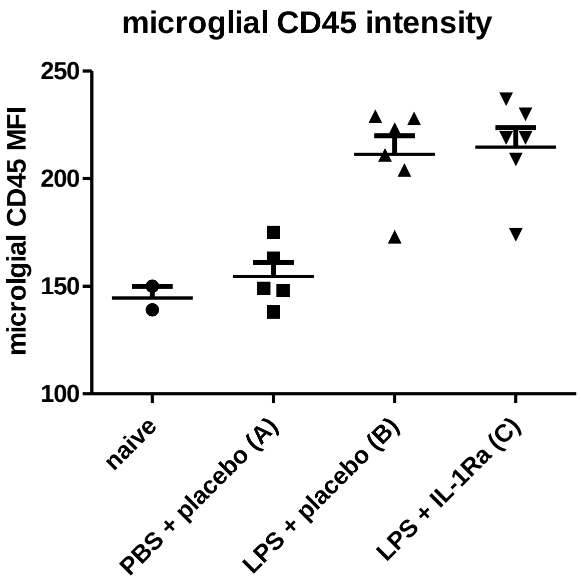

Microglial CD45 intensity was similarly increased after LPS ic challenge in IL-1Ra and placebo-treated mice

B) The fact that central LPS drives a mild peripheral IL-1a response appears to be the justification for assessing the role of systemically applied anti-IL-1 IgG and IL-1ra to assess the role of circulating IL-1a in the LPS induced PMN recruitment in Figure 3C. This seems valid to test, but this seemed unlikely and was perhaps less pressing than testing whether centrally applied IL-1ra/anti-IL-1 IgG block the PMN

European Journal of Immunology

recruitment and whether blocking IL-1b would do likewise. The authors also indicate that the IL-1a response is absent in platelet depleted animals and this data might be shown?
Response: As described above in response to reviewer 1 we now include new data showing that IL-1Ra administered ic significantly reduces neutrophil infiltration to the brain in response to LPS (Fig 3D in revised manuscript). Platelet data are now shown in supplemental figure 2

Given that it is one of the conclusions of the abstract that IL-1 alpha is the dominant IL-1 form in brain innate immunity and this is implied in 2 of the 3 figures in this short communication, I would suggest that the authors should probably demonstrate/refute the contribution of IL-1b to the LPS-induced neutrophil infiltration to the brain.
Response: As stated above, we acknowledge in the manuscript that a role for IL-1beta cannot be entirely excluded despite the magnitude of neutrophil reduction being similar in IL-1alpha and IL-1alpha/beta KO mice. However, our recent data in other models of CNS injury has also shown that IL-1alpha is the dominant early IL-1 ligand driving inflammatory responses in the brain[4, 5]. Currently IL-1beta KO mice are unavailable to us and we would have reservations about centrally applying large doses of blocking antibody given that microglia will be activated via Fc receptors to both control and anti-IL-1 treatments thus confounding interpretation of data.

Supplementary figure 2: Given that the authors take the trouble to investigate whether IgG and IL-1ra do not enter the brain parenchyma, they should show these data at sufficient resolution to examine whether there really is no IgG in the brain; supplementary figure 2A is at very low power, is insufficiently labelled and (if these pictures are intended to demonstrate lack of IgG labelling), they should demonstrate a positive control, in which they predict blood brain barrier breakdown and in which IgG labelling is successfully demonstrated. The addition of IL-1ra labelling at the site of craniotomy (C) is useful makes the lack of labelling in B credible and the same approach might be used for IgG.
Response: We understand the referee's comment about resolution of the IgG images. We did re-examine these tissue sections and because there is no IgG evident at all we could not show with any greater clarity the absence of IgG even at higher magnification. However, as suggested by the referee, we have shown a positive control image (supplemental figure 3B) which demonstrates IgG accumulation in the brain after middle cerebral artery occlusion, an experimental model of stroke in mice that causes overt BBB permeability.

Minor points:
Reference 13 is incomplete (no year)

Response: We have corrected the reference

References

1. Rummel, C., Inoue, W., Poole, S. & Luheshi, G.N. Leptin regulates leukocyte recruitment into the brain following systemic LPS-induced inflammation. *Mol Psychiatry* **15**, 523-534 (2010).

2. Denes, A. *et al.* Central and haematopoietic interleukin-1 both contribute to ischaemic brain injury in mice. *Disease Models & Mechanisms* **6**, 1043-1048 (2013).

3. Boutin, H. *et al.* Role of IL-1Î± and IL-1Î² in Ischemic Brain Damage. *The Journal of Neuroscience* **21**, 5528-5534 (2001).

4. Luheshi, N., Kovacs, K., Lopez-Castejon, G., Brough, D. & Denes, A. Interleukin-1alpha expression precedes IL-1beta after ischemic brain injury and is localised to areas of focal neuronal loss and penumbral tissues. *J Neuroinflammation* **8**, 186.

5. Greenhalgh, A.D. *et al.* Interleukin-1 receptor antagonist is beneficial after subarachnoid haemorrhage in rat by blocking haem-driven inflammatory pathology. *Disease Models & Mechanisms* **5**, 823-833 (2012).

Second Editorial Decision – 19 September 2014

Dear Dr. McColl,

Thank you for submitting your revised manuscript ID eji.201444748.R1 entitled "Requirement for interleukin-1 in driving brain inflammation reveals tissue-specific mechanisms of innate immunity" to the European Journal of Immunology.

Your manuscript has been re-reviewed and the comments of the referees are included at the bottom of this letter. Although the referees have recommended publication, some revisions to your manuscript have been requested. Therefore, I invite you to respond to the comments of the referee and revise your manuscript accordingly.

You should also pay close attention to the editorial comments included below and in the attached file.

If the revision of the paper is expected to take more than three months, please inform the editorial office. Revisions taking longer than six months may be assessed by new referee(s) to ensure the relevance and timeliness of the data.

Once again, thank you for submitting your manuscript to European Journal of Immunology. We look forward to receiving your revision.

Yours sincerely,
Laura Soto Vazquez

on behalf of Prof. Britta Engelhardt

Editorial Office
European Journal of Immunology
e-mail: ejied@wiley.com
www.eji-journal.eu

Peer review correspondence

European Journal of Immunology

****

Reviewer: 1

Comments to the Author
The authors have satisfactorily addressed my comments

Reviewer: 2

Comments to the Author
The authors have made several revisions to the manuscript, which have improved it. However they have not fully addressed one of the major points I had raised:

The authors contention that IL-1 alpha has an important role in LPS-induced neutrophil recruitment to the brain is supported by the IL-1 alpha -/- data in figure 2, but their contention that alpha is the predominant form are insufficiently supported. Their reasoning that since the extent of reduction in neutrophil infiltration in alpha knockouts versus alpha/beta knockouts is similar, that this likely means that IL-1 alpha is the predominant form is not unreasonable but given the residual neutrophil infiltration and the scope for compensation in inflammatory knockouts, it does not exclude a role for IL-1b (and IL-1b is known to be sufficient for neutophil infiltration).

The authors have not performed similar experiments in IL-1b knockout mice (because the are currently unavailable to them) and thus cannot reject the idea that IL-1b also contributes. They state that they cannot exclude a role for IL-1 beta, but thereafter in the paper, they continue to refer to IL-1 alpha as the predominant form: from that point forward they phrase their results and label their figures as if they are specifically targeting IL-1 alpha when they use IL-1 receptor antagonist. In addressing the referee’s request to include direct icv application of an IL-1 blocker to demonstrate IL-1’s role in the neutrophil infiltration the authors did not take the opportunity to separately target IL-1 alpha and beta and instead chose to use IL-1ra which does not show IL-1 alpha/beta specificity. Therefore they should not use the terms central and peripheral IL-1 alpha inhibition. They have applied central and peripheral IL-1 inhibition. They have not taken the opportunity to address whether IL-1b plays any role in LPS-mediated neutrophil infiltration and their statement that IL-1 alpha is a significant contributor to this is sound, but the evidence for their contention that it is the predominant IL-1 isoform in this process remains circumstantial. No direct comparison between the roles of IL-1 alpha and IL-1 beta has been made.
The results section for figure 2 needs to make this more clear and to temper its language accordingly and the text accompanying figure 3 needs to drop many of its references to IL-1 alpha and replace them with IL-1 since there is no specific targeting of IL-1 alpha in those experiments.

European Journal of Immunology

On manuscript page 6 the authors acknowledge that they cannot entirely exclude a role for IL-1b. They should also add to this sentence that IL-1b is known to be sufficient to induce cxcl1 and neutrophil infiltration in the brain. This should be made explicit elsewhere when the authors argue that IL-1 alpha is the predominant IL-1 isoform.

Notwithstanding this, the discussion, and indeed the motivations for the entire study, do not weigh heavily on this distinction between alpha and beta and focus more on the brain-specific reliance on IL-1 with respect to peripheral organs. The study makes the important point that we might therapeutically target IL-1 to mitigate neuroinflammatory responses during insults such as stroke without significantly compromising normal peripheral immune system function.

Second revision – authors' response – 30 September 2014

Reviewer: 1

Comments to the Author
The authors have satisfactorily addressed my comments
Response: We thank the referee for their favourable opinion and previous comments

Reviewer: 2

Comments to the Author
The authors have made several revisions to the manuscript, which have improved it. However they have not fully addressed one of the major points I had raised:

The authors contention that IL-1 alpha has an important role in LPS-induced neutrophil recruitment to the brain is supported by the IL-1 alpha -/- data in figure 2, but their contention that alpha is the predominant form are insufficiently supported. Their reasoning that since the extent of reduction in neutrophil infiltration in alpha knockouts versus alpha/beta knockouts is similar, that this likely means that IL-1 alpha is the predominant form is not unreasonable but given the residual neutrophil infiltration and the scope for compensation in inflammatory knockouts, it does not exclude a role for IL-1b (and IL-1b is known to be sufficient for neutophil infiltration).

The authors have not performed similar experiments in IL-1b knockout mice (because the are currently unavailable to them) and thus cannot reject the idea that IL-1b also contributes. They state that they

European Journal of Immunology

cannot exclude a role for IL-1 beta, but thereafter in the paper, they continue to refer to IL-1 alpha as the predominant form: from that point forward they phrase their results and label their figures as if they are specifically targeting IL-1 alpha when they use IL-1 receptor antagonist. In addressing the referee's request to include direct icv application of an IL-1 blocker to demonstrate IL-1's role in the neutrophil infiltration the authors did not take the opportunity to separately target IL-1 alpha and beta and instead chose to use IL-1ra which does not show IL-1 alpha/beta specificity. Therefore they should not use the terms central and peripheral IL-1 alpha inhibition. They have applied central and peripheral IL-1 inhibition. They have not taken the opportunity to address whether IL-1b plays any role in LPS-mediated neutrophil infiltration and their statement that IL-1 alpha is a significant contributor to this is sound, but the evidence for their contention that it is the predominant IL-1 isoform in this process remains circumstantial. No direct comparison between the roles of IL-1 alpha and IL-1 beta has been made.

The results section for figure 2 needs to make this more clear and to temper its language accordingly and the text accompanying figure 3 needs to drop many of its references to IL-1 alpha and replace them with IL-1 since there is no specific targeting of IL-1 alpha in those experiments.

Response: We have modified and tempered the language throughout the manuscript as advised by the reviewer. This includes removing some specific references to IL-1α in the Abstract, Results and Figure Legends sections referring to Fig 2 and 3. We have amended the text within Fig 3 to only refer to IL-1 in general.

On manuscript page 6 the authors acknowledge that they cannot entirely exclude a role for IL-1b. They should also add to this sentence that IL-1b is known to be sufficient to induce cxcl1 and neutrophil infiltration in the brain. This should be made explicit elsewhere when the authors argue that IL-1 alpha is the predominant IL-1 isoform.

Response: We have amended the section on p6 to read "However we cannot entirely exclude a role for IL-1β particularly given that intracerebral IL-1β administration or overexpression is capable of inducing neutrophil infiltration to the brain and associated chemokines[17, 18]"

Notwithstanding this, the discussion, and indeed the motivations for the entire study, do not weigh heavily on this distinction between alpha and beta and focus more on the brain-specific reliance on IL-1 with respect to peripheral organs. The study makes the important point that we might therapeutically target IL-1 to mitigate neuroinflammatory responses during insults such as stroke without significantly compromising normal peripheral immune system function.

Response: We are pleased that the reviewer has identified that the general dependence of the brain on IL-1 (regardless of specific ligand) versus systemic tissues is the most important conclusion of the study, particularly given the therapeutic implications.

Peer review correspondence

European Journal of Immunology

Third Editorial Decision – 14 October 2014

Dear Dr. McColl,

It is a pleasure to provisionally accept your manuscript entitled "Requirement for interleukin-1 to drive brain inflammation reveals tissue-specific mechanisms of innate immunity" for publication in the European Journal of Immunology. For final acceptance, please follow the instructions below and return the requested items as soon as possible as we cannot process your manuscript further until all items listed below are dealt with.

Please note that EJI articles are now published online a few days after final acceptance (see Accepted Articles: http://onlinelibrary.wiley.com/journal/10.1002/(ISSN)1521-4141/accepted). The files used for the Accepted Articles are the final files and information supplied by you in Manuscript Central. You should therefore check that all the information (including author names) is correct as changes will NOT be permitted until the proofs stage.

We look forward to hearing from you and thank you for submitting your manuscript to the European Journal of Immunology.

Yours sincerely,
Karen Chu

on behalf of Prof. Britta Engelhardt

Dr. Karen Chu
Editorial Office
European Journal of Immunology
e-mail: ejied@wiley.com
www.eji-journal.eu
